# 

**Published:** 1894-12

**Authors:** 


### CONTENTS.

ORIGINAL COMMUNICATIONS—

"Mixed Infection"—Report of Cases. By M. B. Hutchins M.D., Atlanta Ga...... 577
Uterine Cancer. By George R. West, B.S., M.D., Chattanooga, Tenn.............. 580
Early Operation in Appendicitis, with a Case. By J. McFadden Gaston, M.D., Atlanta, Ga.................. 587
The History of Medicine and Surgery in Georgia. By Luther B. Grandy, M.D., Atlanta, Ga.................. 597
The Aseptic and Antiseptic Treatment of Wounds. By William B. Crawford, M.D., Augusta, Ga.................. 603

SOCIETY REPORTS—

Augusta Academy of Medicine.................. 614

EDITORIAL—

Serum Therapy for Diphtheria.................. 620
The Medical Bill.................. 623
About Bills in the Hands of a Collection Agency.................. 623

SELECTIONS AND ABSTRACTS—

Surgery.................. 625
Obstetrics and Gynecology.................. 630
MEDICAL ITEMS.................. 633
BOOK REVIEWS.................. 635
ADVERTISERS' INDEX TO ADVERTISEMENTS.................. xiv
CLASSIFIED INDEX TO ADVERTISEMENTS.................. xxxv
MEDICAL ITEMS—Continued..................x, xi, xii, xiii

